# Anterior cruciate ligament injury incidence in male and female soccer players: A longitudinal study over six consecutive seasons

**DOI:** 10.1002/ksa.70046

**Published:** 2025-09-22

**Authors:** Alfred Ferré‐Aniorte, Ignasi Bolibar, Ramón Cugat, Eduard Alentorn‐Geli

**Affiliations:** ^1^ Universitat Autònoma de Barcelona Cerdanyola del Vallès Spain; ^2^ Fundación García Cugat Barcelona Spain; ^3^ Instituto Cugat Hospital Quironsalud Barcelona Barcelona Spain; ^4^ Mutualidad de Futbolistas Españoles Barcelona Spain

**Keywords:** anterior cruciate ligament injuriers, epidemiology, incidence, soccer, youth sports

## Abstract

**Purpose:**

The aim of this study was to describe the anterior cruciate ligament (ACL) injury incidence differences depending on sex and age‐related categories in a large cohort of soccer players over six consecutive seasons.

**Methods:**

This study was designed as a retrospective descriptive epidemiological study. All soccer players in a specific geographical area who sustained an ACL injury across six consecutive seasons were included in this analysis. ACL injury incidence was calculated by sex and age‐related category as a percentage of all registered soccer players in the region. Additionally, soccer participation evolution, ACL injury incidence evolution, and monthly ACL injury distribution were studied. Group, seasonal, and monthly differences were analysed using chi‐square tests.

**Results:**

Between the 2016–2017 and the 2021–2022 seasons, 3381 ACL injuries were registered from a total of 782,856 player‐seasons. ACL injury incidence was 0.43%. Female soccer players showed 2.79 times higher injury incidence than male players, with overall rates of 1.06% in females and 0.38% in males (*p* < 0.001). However, female players only showed higher ACL injury incidence than males in age groups older than 14 years. ACL injury incidence increased over the six seasons studied only in the male group. October and January were the months with the highest number of ACL injuries, with no significant differences in monthly distribution between sexes.

**Conclusions:**

Female soccer players showed higher ACL injury incidence than males, particularly in age groups older than 14 years. October and January were identified as the months with the highest injury incidences regardless of sex. Additionally, a rising injury incidence was observed in male players, a trend not seen in females.

**Level of Evidence:**

Level III, retrospective comparative study.

**Abbreviations**
ACL: anterior cruciate ligament
CI: confidence interval
FIFA: Fédération Internationale de Football Association
IR: incidence rate
MRI: magnetic resonance imaging
RR: relative risk
SPSS: Statistical Package for the Social Sciences
U: under (used in age‐related categories, e.g., U‐19)

## INTRODUCTION

Anterior cruciate ligament (ACL) injury is one of the most severe injuries related to soccer in terms of time to return to play [11]. After an ACL reconstruction, return to sport is influenced by several physical and contextual factors [3]. Eighty‐one percent of athletes are able to return to sport, with only 55% returning to competitive level. This percentage increases when studying only younger athletes aged 6–19 years, with 92% of them returning to any sport and 81% returning to competitive sport [45]. The analysis of knee injuries, including ACL tears, meniscal tears, or cartilage lesions is also paramount, since major knee injuries have been described as one of the major predisposing factors for knee osteoarthritis [44].

Among all sports, soccer showed one of the highest ACL injury risks, with an incidence rate (IR) of 3.67 (95% confidence interval [CI] 2.61–5.27) [17]. Several studies have also compared the ACL injury incidence between male and female soccer players [17, 32, 36, 41]. The general conclusion is that female soccer players present a greater risk of ACL injury compared to male players, with relative risks (RRs) ranging from 2.67 to 5.51 [17, 32, 36, 41]. The reasons related to such difference are yet to be clarified. However, some authors pointed out that the cause of such disparity may be multifactorial, including anatomical factors such as increased Q angle [25] and pelvic width [34], decreased notch width relative to the ACL size [40], and joint laxity; [9] hormonal differences; [35] and neuromuscular factors such as the hamstring‐to‐quadriceps peak torque [21].

One of the limitations of the current knowledge is the lack of investigations studying the relation between age and ACL injury incidence [43]. In this regard, Astur et al. observed higher incidence of ACL injuries in the under (U)−20 age category compared to U‐13, U‐15 and U‐17 age groups [5]. However, despite the evidence of sex‐related differences in ACL injury incidence, the influence of age in such disparities remains unclear.

Therefore, this study aims to analyse the ACL injury incidence across different age‐related categories and examine how these patterns differ between male and female soccer players over six consecutive seasons.

We hypothesised that female soccer players will show a higher ACL injury incidence compared to male players, and that this difference will vary depending on the age‐related category.

## METHODS

### Study design

This study was designed as a retrospective descriptive epidemiological study.

### Registry characteristics

All the data involved in this study was extracted from the ongoing injury register of the Catalan delegation of the Spanish Soccer Player's Insurance. All soccer players from Catalonia (an 8‐million people autonomous region at the north‐eastern corner of Spain [22]) that plays under the Catalan Soccer Federation must be affiliated with the Catalan delegation of the Spanish Soccer Player's Insurance. This private medical insurance centralises all injury‐related medical care for licensed soccer players in Catalonia, ensuring comprehensive injury registration. The medical team of the insurance registers each injury occurred that needed medical assistance at an internal database with a specific code as part of their daily clinical practice. For the purpose of this study, all soccer players with the code for an ACL injury were identified and exported to a separate database in order to perform the required analysis.

### Ethics

This study was accepted by the Ethics Committee of the Quironsalud Group with code W‐ACLR. All subjects consented the use of their data for epidemiological and sinistrality analysis purposes.

### Patients

This epidemiological study included all the soccer players under the Catalan Soccer Federation that sustained an ACL injury within six consecutive seasons, from the season 2016–2017 to the season 2021–2022. Each season comprised all soccer‐related activities occurred between 1 September and 1 June including practices, matches, and conditioning sessions. The Catalan Soccer Federation oversees all regional soccer leagues up to the first four professional divisions, which are managed by other organisations. Accordingly, the sample of the soccer players included in this study consists mainly of amateur players, with some from semi‐professional teams.

### Variables

The main variable of this study was the cumulative incidence of ACL injuries. The data from the Catalan Soccer Player's Insurance register was accessed to determine the number of injured players in the given period of time. Diagnostic procedures were consistent across the medical team. Each injury was diagnosed through a clinical examination performed by a sports medicine physician or a specialised orthopaedic surgeon, followed by confirmation with magnetic resonance imaging (MRI).

ACL injuries were included regardless of their severity, treatment method (surgical or non‐surgical), or whether they were a first injury or an ACL reinjury. The authors also recorded the date of the injury and the age of the player at the time of the injury.

The total number of soccer players was obtained from the seasonal report of the Catalan Soccer Federation [13]. The total number of soccer players was extracted regarding each sex and pre‐defined age‐related category: Under‐6, Under‐8, Under‐10, Under‐12, Under‐14, Under‐16, Under‐19 and Over‐19.

### Statistical analysis

All ACL injuries registered were included in a separate database with information regarding day and month of the injury, the season, the age‐related category at the time of the injury, and the sex. ACL cumulative incidence was calculated as the number soccer players that sustained an ACL injury divided by the total number of soccer participants, and presented as number of injuries per 100 soccer players and season.

Seasonal variation in the number of soccer players was defined as the percentage of increase or decrease in the number of players compared with the previous season.

Additionally, 2019–2020 and 2020–2021 seasons were excluded from the monthly ACL injury distribution due to the temporary restrictions in soccer participation due to the COVID‐19 pandemic.

Chi square tests were performed in order to establish sex differences between age‐related categories, to analyse the monthly distribution of ACL injuries and to compare ACL injury incidences between 2016–2017 and 2021–2022 seasons.

The statistical analysis was conducted using the SPSS statistical software (SPSS Inc. Released 2006. SPSS for Windows, Version 15.0. Chicago, SPSS Inc.). A significance level of 0.05 was stated as statistically significant over the analysis performed.

## RESULTS

From the 2016–2017 to the 2021–2022 season, a total of 3381 injuries were registered over 782,856 player‐seasons. Female players accounted for 599 of the total number of injuries (17.72%), while only representing 56,530 player‐seasons (7.22%) of the total.

### Soccer participation

**Figure 1:**
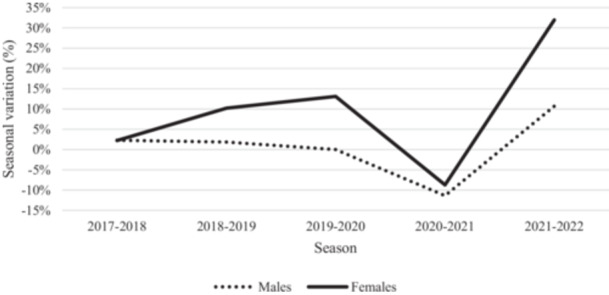
Seasonal variation in the number of soccer players.

The number of soccer players increased over the six included seasons from 127,927 soccer players during the 2016–2017 season up to 134,872 during the 2021–2022 season. A higher increase has been observed between 2016 and 2022 seasons in female soccer players compared to male soccer players (Figure 1). Additionally, a notable decrease was observed in both sexes during the 2020–2021 season, likely due to the restrictions implemented during the COVID‐19 pandemic.

### Injury incidence

The overall ACL injury incidence was 0.43 per 100 soccer players and season (Table 1). When analysed by sex, female soccer players showed a significantly higher injury incidence compared to male soccer players, representing 1.06% and 0.38%, respectively (*p* < 0.001). Accordingly, female players showed an overall ACL injury incidence 2.79 times higher than male players.

**Table 1:** ACL injury incidence across age categories and sex.

	ACL injuries		Licences		Seasonal cumulative incidence (%)			*p*‐Value
	Males	Females	Males	Females	Males	Females	Total	
Over‐19	1487	316	124,696	15,844	1.19	1.99	1.28	<0.001
Under‐19	822	167	105,292	9726	0.78	1.72	0.86	<0.001
Under‐16	351	103	110,526	9023	0.32	1.14	0.38	<0.001
Under‐14	94	11	127,085	9692	0.07	0.11	0.08	0.180
Under‐12	22	2	125,005	7675	0.02	0.03	0.02	0.647
Under‐10	6	0	90,347	3577	0.01	0.00	0.01	1.000
Under‐8	0	0	41,031	961	0.00	0.00	0.00	‐
Under‐6	0	0	2344	32	0.00	0.00	0.00	‐
Total	2782	599	726,326	56,530	0.38	1.06	0.43	<0.001

Abbreviation: ACL, anterior cruciate ligament.

**Figure 2:**
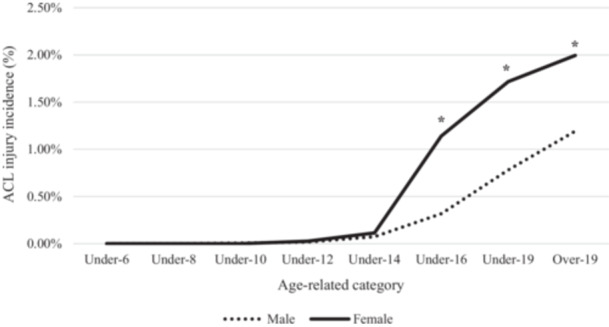
Sex variations in ACL injury incidence depending on the age categories. *Statistically significant difference (*p* < 0.05). ACL, anterior cruciate ligament.

Between‐sex differences were not constant across all age‐related categories. No statistical differences were found between male and female players in ACL injury incidence in Under‐14 category and younger, while ACL injury incidences were significantly different in the Under‐16, Under‐19 and Over‐19 categories (Table 1). The injury incidence in female players was 3.56 times higher than in male players in the Under‐16 category, 2.21 times higher in the Under‐19 category, and 1.67 times higher in the Over‐19 category (Figure 2).

### ACL injury incidence evolution

**Figure 3:**
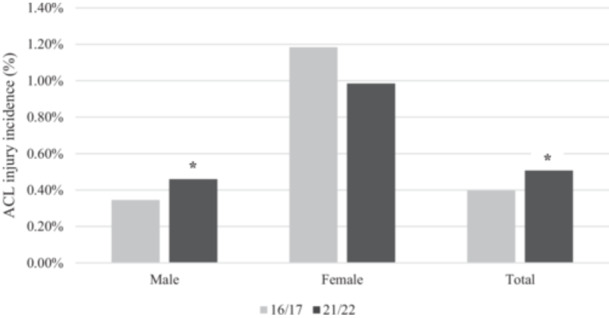
ACL injury differences from 2016/2017 to 2021/2022 seasons. *Statistically significant difference (*p* < 0.05). ACL, anterior cruciate ligament.

When comparing ACL injury incidence between 2016–2017 and 2021–2022 seasons, male players showed a significant increase from 0.35% to 0.46% (*p* < 0.001). This trend was not observed in female players, whose incidence decreased from 1.18% to 0.98% over the same two studied seasons. Overall, the global ACL injury incidence increased significantly from 0.40% to 0.51% (*p* < 0.001) (Figure 3).

### Monthly ACL injury distribution

**Figure 4:**
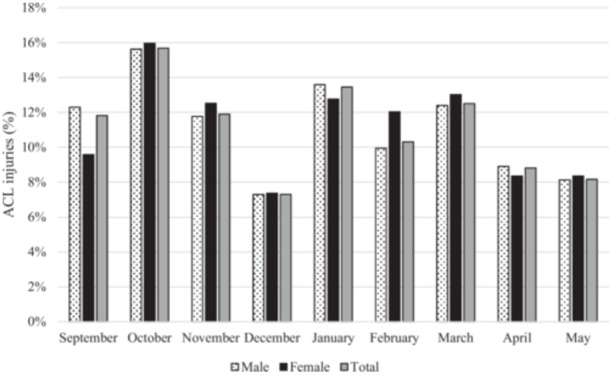
Monthly ACL injuries distribution. ACL, anterior cruciate ligament.

The number of ACL injuries significantly changed depending on the month of the season (*p* < 0.001). The analysis of the monthly distribution of the ACL injuries showed that October was the month with the highest number of ACL injuries (15.7% of the total) followed by January and March with 13.5% and 12.5% respectively (Figure 4). No statistical differences were observed in injury distribution between male and female soccer players (*p* = 0.847).

## DISCUSSION

The main finding of this study is that female soccer players showed a significantly higher ACL injury incidence than male soccer players. However, such difference was not constant across all age‐related categories, with the largest differences observed in the Under‐16 category, while no differences were found in the Under‐14 category and younger.

Female soccer participation has risen notably in recent years. According to the Fédération Internationale de Football Association (FIFA), the number of women playing soccer has grown by up to 24% since 2019 [14]. These findings align with those presented in this study, with 53.50% increase in female soccer participation from the 2016–2017 to the 2021–2022 season. This surge in female soccer players is likely to be associated with a corresponding increase in injuries, particularly those more prevalent in female soccer, such as ACL injuries.

The difference between male and female ACL injury rates has been corroborated by several studies and reviews [32, 36, 48]. In a recent systematic review, Bram et al. reported a per‐season risk of ACL injury of 1.21% for female and 0.39% for male soccer players [7]. These results agree with those presented in the present study with ACL injury incidences of 0.38% and 1.06% for male and female soccer players, respectively. However, the specific causes of such differences are still being studied.

Several risk factors have been previously described [1, 20, 21, 27, 30]. Anatomical differences between male and female athletes have been linked to increased ACL injury risk, although the magnitude of their effect remains controversial [1]. Female athletes have presented smaller femoral intercondylar notch volume which may impinge the ACL increasing the injury risk [23, 30]. Meanwhile, increased posterior slope of the lateral tibial plateau articular cartilage surface has been also linked to increased ACL injury risk among female athletes [42]. Tissue tensile properties have also been studied, with females showing fewer fibrils per area [1, 19].

Hormonal concentration modifications across the menstrual cycle have been hypothesised to play an important role in ACL injury risk among female athletes [1, 21]. Higher ACL laxity has been observed during ovulation, when estradiol concentrations are higher compared to the menstruation phase, when estradiol levels are at its lowest [28]. However, higher relaxin levels have been observed during menstrual cycle days, suppressing local ACL collagen production [35] and therefore influencing the injury risk [9], as specific relaxin receptors have been found in female ACL tissue but not in males [10].

Despite the predictive potential of hormonal and anatomical factors, the unmodifiable nature of most of the previously described risk factors has pushed the scientific community to investigate further potential contributors that could be addressed in order to minimise ACL injury incidence. Accordingly, several neuromuscular risk factors have been described with a higher potential for modification [21]. Dynamic knee valgus and an increased ground reaction force during jumping tasks have been linked to higher risk for ACL injury among female athletes [21]. Additionally, female athletes have shown slower hamstrings pre‐activation and early quadriceps activation before dynamic stability tasks [47] and a more erected posture when landing with lower hip and knee flexion angles [26] leading to higher ACL injury rates [1, 21].

In summary, ACL injury risk is multifactorial with anatomical, hormonal and neuromuscular elements affecting it [46]. The main results of this study support the role of biological maturation changes experienced during puberty as key factors influencing ACL injury incidence. The highest between‐sexes differences in ACL injury incidence were observed at the Under‐16 age‐related category, which includes athletes aged between 14 and 16 years old. At that point, female soccer players presented an ACL injury incidence 3.56 times higher than male soccer players. During this period, female athletes are at the end of its biological maturation while males are generally still undergoing the process [29, 31].

Another important finding of the current study is the progressively increase in ACL injury incidence depending on the age‐related category. Over‐19 group presented the highest ACL injury incidence in both male and female soccer players. These results agree with those presented recently by Astur et al. describing higher ACL injury incidences among Under‐20 Brazilian soccer players compared to younger categories [5]. Older soccer players may be subjected to higher internal and external workloads as well as higher performance demands, increasing hours of athletic exposure and subsequently increasing the absolute number of ACL injuries. However, the disparities in the injury incidence among the youngest age‐related categories reported in this study should be interpreted with caution. Given the limited number of events recorded in the Under‐14 and younger age‐related groups, the statistical estimates for these categories may be less reliable compared to older groups.

In order to address the current number of ACL injuries, several injury prevention strategies have been developed and implemented with promising results [8]. The performance of soccer‐specific injury prevention programmes such as the FIFA 11+ has shown an important reduction in the likelihood of sustaining an ACL injury in both male and female soccer players [38, 39]. Additionally, the goal of reducing the global number of ACL injuries in soccer players may involve multiple benefits for both the athletes and the society. Arundale et al. reported shorter career length after ACL reconstruction compared to healthy controls in major league soccer players [4]. On the other hand, Ross et al. predicted an annual savings of 1.5 million Australian dollars after a hypothetical deployment of a national injury prevention strategy in Australia [37]. Furthermore, the presence of an intraarticular knee injury, not only limited to the ACL, has been described as the major predisposing factor for the development of future knee osteoarthritis [16, 44].

The results presented in this study showed an overall significant increase in the ACL injury incidence during the past six seasons as shown in previous similar studies [2]. Such increase is primarily lead by the significant increase observed in the male group, while no significant differences were observed in the female group. Such difference could be explained by a rising concern about the increased injury incidence registered in female athletes [32] which may have induced coaches, medical staff, and athletes to improve its compliance towards injury prevention programmes. Additionally, recent epidemiological studies have reported a shift from ligament injuries to muscle injuries in elite female soccer players [18], which could be explained by the increased intensity [6] and professionalisation of female soccer leagues.

Finally, a significant difference in ACL injury distribution was observed depending on the month of the season. October and January were the periods with higher ACL injury concentrations, representing 15.7% and 13.5% respectively. Seasons 2019–2020 and 2020–2021 were excluded from such analysis due to the restrictions in soccer participation derived from the lockdown policy followed by the national government during the COVID‐19 pandemic. The observed increase in ACL injuries may be explained by the fact that both months represent a return to competition point after a break period in which, excluding professional and semi‐professional teams, few soccer activities are performed. Such short‐term modifications in workload have been related to higher injury incidences in male rugby union and soccer players [12, 24]. The results presented in this study suggest that female soccer players could be influenced in a similar way, aligning with previous epidemiological studies [33].

The present study has several limitations. First, it does not provide incidence values based on hours of athletic exposure, which makes it impossible to perform a Cox regression analysis, as individual exposure time and follow‐up data were not available. Given the nature of the internal database used for this study, only injured players were recorded with no further control over the hours each player spent in practice sessions or matches. This methodological limitation makes the results sensitive to temporal variations in soccer‐related activities. However, the registry of specific athletic exposure values would have made it impossible to reach the sample size included. Another limitation could be the lack of discrimination between first‐time ACL injuries and reinjuries. Although injury risk can be affected by the presence of a previous ACL injury, the present analysis could provide a higher external validity approach on the expected per‐season incidence of ACL injury independently of the presence or absence of previous ACL lesions. This study has also several strengths. First, it contains 3.381 ACL injuries across six consecutive seasons, becoming one of the largest epidemiological studies in the field of ACL injuries in soccer players and providing the consistency needed for performing sex and age sub‐analysis simultaneously. All injuries were registered at the same medical institution, allowing for a more homogenic criteria at the time of injury diagnosis. Finally, it includes all soccer participants in a certain geographical region including mostly amateur but also a few semi‐professional teams. Previous incidence studies have been performed mostly over professional or semi‐professional soccer players, which may have access to a well‐stablished medical infrastructure in its daily practice. However, such population only represents 0.042% of the overall soccer players [15]. Unfortunately, the majority of the amateur soccer community does not have day‐to‐day access to medical services. Accordingly, the present study provides representative data for real‐world conditions, offering a close reflection of actual athletic settings.

## CONCLUSION

The results of this study indicates that female players have a significantly higher injury incidence, particularly in the Under‐16 and older categories and that such incidence increases with age. The results also highlight October and January as the months with the highest number of recorded ACL injuries as well as a tendency towards an increase in injury risk in male players not seen in females. As the number of male but particularly female soccer players continue to rise, it is crucial to implement injury preventive strategies, particularly for the population groups and time periods with higher injury risk.

## AUTHOR CONTRIBUTIONS

The author Alfred Ferré‐Aniorte was involved in the study design, data acquisition, statistical analysis, and writing of the final manuscript. The author Ignasi Bolibar collaborated in the initial design, statistical analysis, and manuscript correction. The author Eduard Alentorn‐Geli was involved in study design, data acquisition, statistical analysis, and manuscript writing. Finally, Ramón Cugat oversaw the coordination and the study design and collaborated in data acquisition. All authors approved the final draft of this manuscript.

## CONFLICT OF INTEREST STATEMENT

The authors declare no conflicts of interest.

## ETHICS STATEMENT

This study was approved by the Ethics Committee of the hospital (CEIm Grup Hospitalari Quironsalud‐Catalunya) with the code W‐ACLR. All subjects consented to use their data for epidemiological and sinistrality analysis purposes.

## Data Availability

The data regarding injured subjects related to this study are available from the corresponding author upon reasonable request. Data regarding soccer participation used in this study are available as open data and can be found at the official website of the organisation: https://www.fcf.cat/assamblea.
